# Superior survival benefits of Radical Prostatectomy than External Beam Radiotherapy in aging 75 and older men with high-risk or very high-risk Prostate Cancer: a population-matched study

**DOI:** 10.7150/jca.46069

**Published:** 2020-07-11

**Authors:** Yan Wang, Pan Song, Jiaxiang Wang, Mengxuan Shu, Qingwei Wang, Qi Li

**Affiliations:** 1 Department of Urology, The First Affiliated Hospital of Zhengzhou University, Zhengzhou, 450052, Henan Province, China.; 2 Department of Urology, West China Hospital of Sichuan University, Chengdu, 610000, Sichuan Province, China.; 3 The first Clinical Medical College of Lanzhou University, Lanzhou, 730000, Gansu Province, China.

**Keywords:** High-risk/very high-risk prostate cancer, radical prostatectomy, external beam radiation, survival, SEER

## Abstract

**Objective:** To evaluate the survival difference of radical prostatectomy (RP) and external beam radiotherapy (EBRT) in elderly men (75 years and older) with high-risk (HR) or very high-risk (VHR) prostate cancer (PCa).

**Methods:** Elderly men diagnosed with HR/VHR PCa from 2004-2015 in the Surveillance, Epidemiology and End Results (SEER) database were identified. Propensity-score matching (PSM) was conducted to balance the covariates; Kaplan-Meier and Cox analysis were performed to evaluate the overall survival (OS) and prostate cancer-specific survival (PCSS).

**Results:** 11698 patients with HR PCa and 4415 patients with VHR PCa were identified and divided into RP and EBRT group. After PSM, 964 patients with HR PCa and 538 patients with VHR PCa were included in each group. The 10-year OS and PCSS of men with HR PCa were 60.1% vs 40.9% and 90.6% vs 83.4%, respectively. The 10-year rate of OS and PCSS in men with VHR PCa were 55.9% vs 33.3% and 82.4% vs 75.6%, respectively. The OS curve of patients with HR PCa revealed that RP was significantly better than EBRT in both overall cohort [HR: 0.533, 95%CI (0.485~0.586), p<0.001] and the matched cohort [HR: 0.703, 95%CI (0.595~0.832), p<0.001]. However, the PCSS curve of patients with HR PCa showed that RP was significantly better than EBRT in overall cohort [HR: 0.453, 95%CI (0.368~0.559), p<0.001] but was similar to EBRT in matched cohort [HR: 0.820, 95%CI (0.552~1.218), p=0.327]. As for patients with VHR PCa, RP was associated with better OS than EBRT whether in overall cohort [HR: 0.520, 95%CI (0.457~0.592), p<0.001] or matched cohort [0.695, 95%CI (0.551~0.876), p=0.002]. The PCSS of RP was significantly better than that of EBRT in overall cohort [HR: 0.538, 95%CI (0.422~ 0.685), p<0.001], but was similar in matched cohort [HR: 0.787, 95%CI (0.510 ~1.214), p=0.281].

**Conclusions:** RP has more survival benefits than EBRT in men aged 75 years and older with HR or VHR PCa.

## Introduction

Prostate cancer (PCa) is the most common malignancy in the male genitourinary system, accounting for about 15% of all malignant tumors in the world. It ranks first in the incidence of male malignancies, and the second in male cancer-related deaths, seriously threatening the lives and health of male patients 1, 2. In 2020, it is estimated that more than 191,930 men in the United States are newly diagnosed with PCa and 33,330 cases of PCa deaths 3. With the widespread use of prostate-specific antigen (PSA), the incidence of PCa and the clinical stage of newly diagnosed PCa have changed dramatically. The proportion of early-stage PCa increased significantly, while the advanced PCa greatly reduced in newly diagnosed cases 4-6. Among the newly diagnosed PCa patients, localized PCa accounts for approximately 80% 7, 8. According to the guideline of National Comprehensive Cancer Network (NCCN) for prostate cancer, the high-risk (HR) PCa is defined as T3a, PSA>20ng/ml, or Gleason Score (GS 8-10), and Very high-risk (VHR) as T3b-4, grade group 5, or >4 core positive grade group 4-5 9. HR/VHR PCa is characterized by recurrences and high cancer-related deaths. Besides, VHR PCa is more aggressive and associated with higher risks of cancer-specific mortality than HR PCa.

Nowadays, the main radical local treatments for HR/VHR PCa are radical prostatectomy (RP) with/without antiandrogen treatment (ADT) and external beam radiation (EBRT). RP is the preferred choice of treatment for specific patients with localized diseases and life expectancy >10 years 10, 11. However, its benefits vary in patients, especially in elderly men. In recent years, with the development of radiotherapy equipment and technology, the side effects of EBRT have remarkably reduced, making EBRT an effective and important treatment for HR/VHR PCa.

Even though many trials have evaluated the benefits of RP and EBRT in HR PCa and have shown that RP seems to have more survival benefits than EBRT. But it is still unclear whether RP remains superior to EBRT in elderly men (75 years and older) with HR PCa, or VHR PCa. The aim of this study was to evaluate the prognostic differences between RP (with or without ADT) and EBRT in elderly men (75 years and older) with localized HR PCa or VHR PCa.

## Materials and Methods

### Data source

The data of this study were derived from the Surveillance, Epidemiology and End Results (SEER) database with the software SEER* STAT. Elderly patients (aged 75 years and older) with localized PCa (cT1-4N0M0) diagnosed from January 1, 2004 to December 31, 2015 were retrospectively identified.

### Inclusion and exclusion criteria

Patients were considered eligible if they met the following criteria: (1) patients with primary localized (cT1-4N0M0) PCa. (2) Patients were 75 years and older at the time of diagnosis. (3) Patients were diagnosed with HR PCa or VHR PCa according to the definition of NCCN guideline. (4) The treatments were RP (with or without ADT) or EBRT.

The following criteria were used for data exclusion: (1) Multiple tumors; (2) Important information such as PSA, GS, and TNM staging was incomplete or missing; (3) The survival status at the end of the follow-up was unclear, or the follow-up time was incomplete.

### Variables and main outcomes

Patients' general information, tumor information, and survival status were collected. The variables included age (75-79, 80-84, ≥85), race (white, black, other races including American Indian and Asian/Pacific Islander), marital status (married, unmarried, divorced or separated), T stage (T1, T2, T3, T4), PSA level ) ≤20 ng/ml, >20 ng/ml), GS (<8, 8-10), treatment (RP, EBRT), survival time, living status (alive or dead) and cancer-specific living status (alive, died for other reasons, died for prostate cancer). The main outcomes of this study were overall survival (OS) and prostate cancer-specific survival (PCSS) in overall cohort and matched cohort.

### Statistical analyses

Chi-square test was used to assess the baseline characteristics between the RP group and EBRT group. Propensity-score matching (PSM) based on the nearest-neighbor matching principle was conducted to balance the covariates in two groups. OS and PCSS curves were constructed with Kaplan-Meier (K-M) analysis, and 5-year and 10-year OS and PCSS were calculated by survival tables. Univariate COX analysis was used for each variable to obtain the relevant parameters of OS and PCSS prognosis. Multivariate Cox analysis was performed on the variable with P<0.1. The effects of various factors on the survival and prognosis were evaluated by hazard ratio (HR) values with 95% confidence interval (95% CI). The above statistical operations were performed with the software of SPSS 25 and Graph prism 7. P < 0.05 was considered statistically significant.

## Results

### Patient characteristics

11698 patients with HR PCa were identified, with a median age of 76 (78-81) years with 1307 patients in RP group and 10391 patients in EBRT group. The median follow-up time is 60 (32-90) months. After PSM, 964 patients were left in each group. The baseline characteristics of two groups in both overall cohort and matched cohort were shown in **Table 1**.

4415 patients with VHR PCa were identified, with 742 patients in RP group and 3673 patients in EBRT group. 538 patients remained in each group after PSM. The baseline characteristics of overall cohort and matched cohort were presented in **Table 2**.

### Survival outcomes

#### 5-year and 10-year OS and PCSS rates

The 10-year OS rate of patients with HR PCa in RP group and EBRT group were 60.1% vs 40.9% (P<0.001) in overall cohort, and 56.3% vs 45% (P<0.001) in matched cohort. The 5-year and 10-year PCSS rate of the RP group and EBRT group were 90.6% vs 83.4% (P<0.001) in overall cohort, and 88.0% vs 87.0% (P=0.136) in matched cohort. The 5- and 10- year OS and PCSS rates in two cohorts were presented in **Table 3**.

The 10-year OS rate of patients with VHR PCa in RP group and EBRT group were 55.9% vs 33.3% (P<0.001) in overall cohort, and 56.9% vs 41.5% (P<0.001) in matched cohort. The 10-year PCSS rate of the RP group and EBRT group was 82.4% vs 75.6% (P<0.001) in overall cohort, and 82.9% vs 79.2% (P=0.073) in matched cohort. These results were also revealed in **Table 3**.

#### Survival curves

##### Survival curves of men with HR PCa

**Figure 1:**
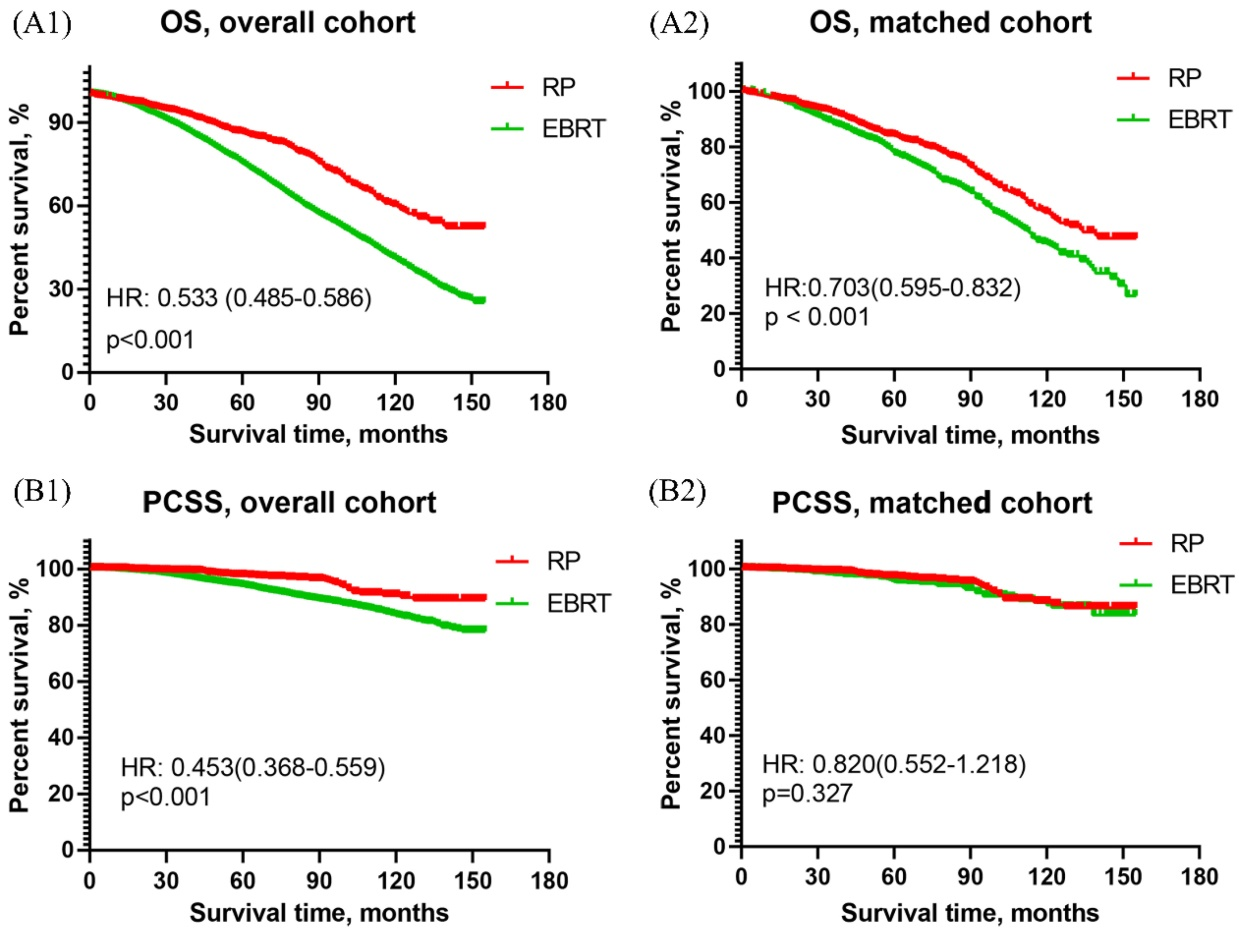
Overall survival and prostate cancer-specific survival curves of RP and EBRT groups for aging 75 and older patients with high-risk prostate cancer. (A1) Overall survival curve of RP and EBRT in overall cohort. (A2) Overall survival curve in propensity matched cohort. (B1) Prostate cancer-specific survival curve of RP and EBRT in overall cohort. (B2) Prostate cancer-specific survival curve of RP and EBRT in propensity matched cohort.

The OS curves of men with HR PCa showed that RP was associated with significantly better OS than EBRT in both overall cohort [HR: 0.533, 95% CI (0.485~0.586), p<0.001] and matched cohort [HR: 0.703, 95% CI (0.595~0.832), p<0.001]. The results were presented in **Figure 1A**. As for the results of PCSS curves, RP was obviously better than EBRT in overall cohort [HR: 0.453, 95% CI (0.368~0.559), p<0.001] but they two were similar in matched cohort [HR: 0.820, 95% CI (0.552~1.218), p=0.327]. The results were shown in **Figure 1B**.

##### Survival curves of men with VHR PCa

**Figure 2:**
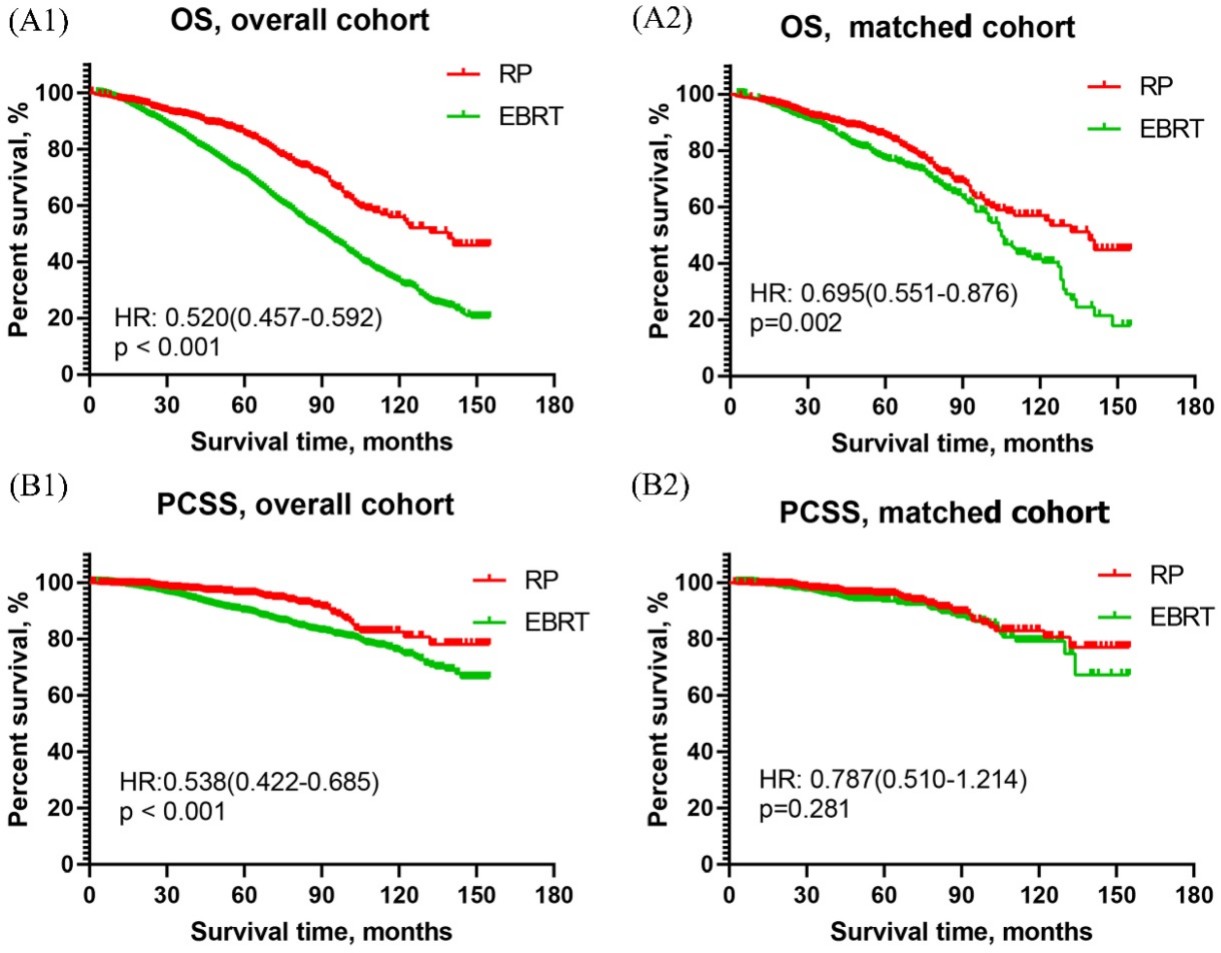
Overall survival and prostate cancer-specific survival curves of RP and EBRT groups for aging 75 and older patients with very high-risk prostate cancer. (A1) Overall survival curve of RP and EBRT in overall cohort. (A2) Overall survival curve in propensity matched cohort. (B1) Prostate cancer-specific survival curve of RP and EBRT in overall cohort. (B2) Prostate cancer-specific survival curve of RP and EBRT in propensity matched cohort.

As shown in **Figure 2A**, the OS curve of men with VHR PCa revealed that RP was apparently better than EBRT in both overall cohort [HR:0.520, 95%CI (0.457 ~0.592), p<0.001] and matched cohort [HR: 0.695, 95% CI (0.551~0.876), p=0.002]. The PCSS curve of men with VHR PCa showed that RP had significantly better PCSS than EBRT in overall cohort [HR: 0.538, 95% CI (0.422~0.685), p<0.001], and had similar results with EBRT in matched cohort [HR: 0.787, 95% CI (0.510~1.214), p=0.281]. This was presented in **Figure 2B**.

#### Multivariate COX analysis for OS and PCSS

Multivariate COX analysis results of the OS and PCSS were presented in **Table 4**. Factors related to the risk of death included age, race, marital status, T-stage, Gleason score, PSA, and treatments. With RP as the reference, The HR and 95%CI of EBRT for overall mortality of men with HR and VHR PCa were 1.62 (1.42~1.86), and 1.55 (1.29~1.87), respectively. The HR and 95%CI of EBRT for cancer-specific mortality of men with HR and VHR PCa were 1.86 (1.35~2.55), and 1.58 (1.12~2.21), respectively.

## Discussion

Age is an important factor that has an impact on the prognosis of HR/VHR PCa and affects the choice of treatments for clinicians and patients. The older patients at diagnosis are more likely to be at high risk and less likely to receive curative local therapies 12. RP (with/without ADT) and EBRT are two main curative local therapies for non-metastatic HR/VHR PCa. Even though many studies have evaluated the survival difference of RP and EBRT and have revealed that RP seems to have more survival benefits than EBRT in men with HR or VHR PCa. However, the benefits seem to be inconclusive for elderly men, especially those aged 75 and older because their life expectancy varies.

A total of 11698 patients aged 75 and older with localized HR PCa and 4415 with VHR PCa were included in our study. After PSM, 1928 patients with HR PCa and 1076 patients with VHR PCa were left. The 10 years OS rate of men with HR PCa in RP and EBRT group were 60.1% vs. 40.9% in overall cohort and 56.3% vs. 45.0% in matched cohort. The 10-year PCSS rate of those in RP and EBRT groups were 90.6% vs. 83.4% in overall cohort and 88.0% vs. 87.0% in matched cohort, respectively. This result was consistent with many studies' findings 13-15. Boorjian et al. 16 analyzed 1,238 patients with localized high-risk PCa who underwent RP and 609 patients receiving EBRT. The10-year PCSS rate was 92% and 88%, respectively (p=0.06). Eifler et al. 17 showed that men who underwent RP had a 10-year PCSS rate of more than 90%. A study 18 analyzed the prognosis of 234 patients over 80 years old who underwent RP surgery. In this study, the 10-year OS rate was 51% and 10-year PCSS was 9.9%, similar to our results. In addition, Bandini et al. 19 analyzed the survival of old patients (>75 years) with T1-2 localized PCa who received local treatments (RP or EBRT). Its results showed that the 10-year prostate cancer-specific mortality in RP and EBRT groups were 4.3% and 6.1%, respectively.

The VHR PCa is more aggressive and is associated with a poorer prognosis for the patients than HR PCa. Pompe et al. 20 found that VHR PCa had obviously worse biochemical recurrence-free survival, metastatic progression-free survival, OS and PCSS rates compared to HR PCa. In our study, the 10-year OS rate of RP and EBRT group were 55.9% vs.33.3% in overall cohort and 56.9% vs 41.5% in matched cohort. The 10-year PCSS rate of two groups were 82.4% vs 75.6% in overall cohort and 82.9% vs 79.2% in matched cohort. As we know, there were few studies evaluating the prognosis differences between RP and EBRT in aging 75 and older patients with VHR PCa. Sundi et al.^21^ analyzed the outcomes of HR PCa and VHR PCa after the treatment of RP, and found that the 5- year all-cause mortality and cancer-specific mortality were 0.033 and 0.007 in patients with HR PCa and 0.006 and 0.045 in those with VHR PCa. Reichard et al. 22 reported that the all-cause mortality rate of RP and radiotherapy for patients with HR PCa and VHR PCa were 9.5% and 8.2%.

The results of K-M analysis showed that men with HR PCa or VHR PCa in RP group were associated with obviously better OS than those in EBRT group no matter in overall cohort or matched cohort**.** This result revealed that RP had more OS benefits than EBRT in aging 75 and older patients with HR PCa and even with VHR PCa. As for the PCSS results in overall cohort, RP group was significantly better than EBRT group in men with HR PCa or VHR PCa. However, the difference between two groups was not significant in matched cohort. Therefore, we could find out that RP had more survival benefits than EBRT in aging 75 and older men with HR PCa and with VHR PCa.

The COX analysis showed that risk factors associated with prognosis in patients with localized high-risk prostate cancer were age, race, marital status, T stage, PSA, Gleason score, and treatment. A similar study 23 found that risk factors for PCa include family history, genetics, age, ethnicity, and tumor characteristics. According to the results of COX analysis, the risk of death in the EBRT group was significantly higher than that in the RP group, which means that the prognosis of EBRP is worse than that of RP. It was consistent with the results of the OS and PCSS curves. Two meta-analyses reported that OS and PCSS of RP were significantly better than those of EBRT in high-risk PCa patients 24, 25. RP seemed to be more likely to reduce the long-term total mortality and prostate cancer-specific mortality in elderly patients with high-risk prostate cancer.

Even though our study analyzed a large sample of patients, there were still some limitations in our study. The limitations of our study were as follows: (1) Our study was still a retrospective analysis in which there were some unavoidable confounders and risk biases. This may have an impact and interference with the results. (2) Even though we analyzed men aged 75 years and older in our study, the large percentages were in 75-79 years old subgroup and only a few percentages of patients were in ≥85 years subgroup. Therefore, our results might not be suitable for all patients aging older than 75 years. (3) Besides the survival benefits, many factors of individuals such as health status (associated with chronic diseases or not, food intake, body mass index, mobility, etc.), patients' willingness, life expectancy, the quality of life after treatments should be fully considered when choosing RP as the main treatment. RP should be performed in the selected patients but not all elderly patients aging 75 and over. (4) Our study only focused on the analysis of survival prognosis, many important clinical outcomes were not analyzed, such as post-treatment comorbidities, quality of life of patients. It is not appropriate to evaluate two treatments only by survival prognosis.

## Conclusion

Among men aged 75 and older with HR PCa and even VHR PCa, RP was associated with more survival benefits than EBRT. However, with the limitations of our study, high-quality studies are needed for future evaluations.

## Figures and Tables

**Table 1:** The baseline characteristics of patients with high-risk prostate cancer

Variables	Overall cohort			Propensity-matched cohort		
	RP (n=1307)	EBRT (n=10391)	P	RP (n=964)	EBRT (n=964)	P
**Age (years)**						
Median (IQR)	76 (75-78)	78 (76-81)		77 (76-78)	79 (78-79)	
**Age, n (%)**						
75-79	1143 (87.5)	6551 (63)	<0.001	806 (83.6)	796 (82.6)	0.837
80-84	137 (10.5)	3131 (30.1)		132 (13.7)	140 (14.5)	
≥85	27 (2.1)	709 (6.8)		26 (2.7)	28 (2.9)	
**Race, n (%)**						
White	1115 (85.3)	8474 (81.6)	<0.001	832 (86.3)	820 (85.1)	0.837
Black	66 (5)	939 (9)		49 (5.1)	51 (5.3)	
Others	116 (8.9)	864 (8.3)		78 (8.1)	86 (8.9)	
Unclear	10 (0.8)	114 (1.1)		5 (0.5)	7 (0.7)	
**Marital status, n (%)**						
Married	1014 (77.6)	7229 (69.6)	<0.001	717 (74.4)	718 (74.5)	0.930
Unmarried	76 (5.8)	634 (6.1)		63 (6.5)	64 (6.6)	
Separated	163 (12.5)	1681 (16.2)		138 (14.3)	131 (13.6)	
Unclear	54 (4.1)	847 (8.2)		46 (4.8)	51 (5.3)	
**T stage, n (%)**						
T1-2	550 (42.1)	9662 (93)	<0.001	550 (57.1)	544 (56.4)	0.783
T3a	757 (57.9)	729 (7)		414 (42.9)	420 (43.6)	
**GS, n (%)**						
<8	545 (41.7)	1994 (19.2)	<0.001	282 (29.3)	276 (28.6)	0.763
8-10	762 (58.3)	8397 (80.8)		682 (70.7)	688 (71.4)	
**PSA (ng/ml)**						
Median (IQR)	7.8 (5.4-12.2)	11.8 (7.1-23.5)		8.0 (5.5-14.3)	9.5 (6.3-16.1)	
**PSA (ng/ml), n (%)**						
<20	1131 (86.5)	6998 (67.3)	<0.001	793 (82.3)	799 (82.9)	0.719
>20	176 (13.5)	3393 (32.7)		171 (17.7)	165 (17.1)	
**Follow-up time (months)**						
Median (IQR)	62 (33-93)	60 (32-89)		58.5 (32-89.8)	62 (33-91)	

IQR, interquartile range; RP, radical prostatectomy; EBRT, external beam radiotherapy; GS: Gleason score; PSA, prostate-specific antigen.

**Table 2:** The baseline characteristics of patients with very high-risk prostate cancer

Variables	Overall cohort			Propensity-matched cohort		
	RP (n=742)	EBRT (n=3673)	P	RP (n=538)	EBRT (n=538)	P
**Age (years)**						
Median (IQR)	76 (75-78)	78 (76-82)		76 (75-78)	76 (75-79)	
**Age, n (%)**						
75-79	638 (86)	2222 (60.5)	<0.001	440 (81.8)	437 (81.2)	0.961
80-84	94 (12.7)	1163 (31.7)		88 (16.4)	90 (16.7)	
≥85	10 (1.3)	288 (7.8)		10 (1.9)	11 (2)	
**Race, n (%)**						
White	639 (86.1)	3070 (83.6)	<0.001	477 (88.7)	466 (86.6)	0.593
Black	38 (5.1)	279 (7.6)		22 (4.1)	28 (5.2)	
Others	60 (8.1)	287 (7.8)		36 (6.7)	38 (7.1)	
Unclear	5 (0.7)	37 (1)		3 (0.6)	6 (1.1)	
**Marital status, n (%)**						
Married	586 (79)	2587 (70.4)	<0.001	417 (77.5)	409 (76)	0.867
Unmarried	37 (5)	218 (5.9)		26 (4.8)	31 (5.8)	
Separated	91 (12.3)	617 (16.8)		75 (13.9)	75 (13.9)	
Unclear	28 (3.8)	251 (6.8)		20 (3.7)	23 (4.3)	
**T stage, n (%)**						0.894
T1-2	159 (21.4)	2989 (81.4)	<0.001	159 (29.6)	161 (29.9)	
T3-4	583 (78.6)	684 (18.6)		379 (70.4)	377 (70.1)	
**GS, n (%)**						
<8	205 (27.6)	128 (3.5)	<0.001	104 (19.3)	105 (19.5)	0.939
8-10	537 (72.4)	3545 (96.5)		434 (80.7)	433 (80.5)	
**PSA (ng/ml)**						
Median (IQR)	8.2 (5.7-12.6)	10.7 (6.8-19.3)		8.4 (5.8-13.7)	9.0 (6.2-14.3)	
**PSA (ng/ml), n (%)**						
<20	671 (90.4)	2804 (76.3)	<0.001	473 (87.9)	483 (89.8)	0.333
>20	71 (9.6)	869 (23.7)		65 (12.1)	55 (10.2)	
**Follow-up time (months)**						
Median (IQR)	54 (27-85)	53 (29-82)		53 (26-81)	56 (31-83)	

IQR, interquartile range; RP, radical prostatectomy; EBRT, external beam radiotherapy; GS: Gleason score; PSA, prostate-specific antigen.

**Table 3:** 5-year and 10-year OS and PCSS for men aging 75 and older with high-risk and very high-risk prostate cancer

Variables		Overall cohort			Propensity-matched cohort		
		RP (%)	EBRT (%)	P	RP (%)	EBRT (%)	P
**High risk PCa**							
OS	5 years	86.2 (84%~88.4%)	75.2 (74.2%~76.2%)	<0.001	84.1 (81.4%~86.8%)	77.3 (74.4%~80.2%)	<0.001
	10 years	60.1 (55.6%~64.6%)	40.9 (39.5%~42.3%)	<0.001	56.3 (50.8%~61.8%)	45 (40.1%~49.9%)	<0.001
PCSS	5 years	97.5 (96.5%~98.5%)	94 (93.4%~94.6%)	0.054	97 (95.6%~98.4%)	95.4 (93.8%~97%)	0.068
	10 years	90.6 (87.5%~93.7%)	83.4 (82%~84.8%)	<0.001	88 (83.9%~92.1%)	87 (83.1%~90.9%)	0.136
**Very high risk PCa**							
OS	5 years	85.3 (82.4%~88.2%)	71.3 (69.5%~73.1%)	<0.001	85.1 (81.6%~88.6%)	76.9 (72.8%~81%)	<0.001
	10 years	55.9 (49.4%~62.4%)	33.3 (30.8%~35.8%)	<0.001	56.9 (49.5%~64.3%)	41.5 (34.2%~48.8%)	<0.001
PCSS	5 years	96.1 (94.3%~97.9%)	89.8 (88.6%~91%)	<0.001	95.9 (93.9%~97.9%)	93.4 (91%~95.8%)	0.131
	10 years	82.4 (76.7%~88.1%)	75.6 (72.9%~78.3%)	<0.001	82.9 (76.4%~89.4%)	79.2 (72.3%~86.1%)	0.073

OS: overall survival; PCSS: prostate cancer-specific survival; RP: radical prostatectomy; EBRT: external beam radiotherapy.

**Table 4:** Multivariate COX analysis for high-risk and very high-risk patients in matched groups

Risk factors	High-risk				Very high-risk			
HR (95%CI)	OS		PCSS		OS		PCSS	
**Age**								
75-79	1	Ref.	1	Ref.	1	Ref.	1	Ref.
80-84	1.44 (1.35~1.54)	< 0.001	1.36 (1.17~1.57)	< 0.001	1.4 (1.26~1.56)	< 0.001	1.21 (0.98~1.48)	0.073
≥85	2.08 (1.86~2.32)	< 0.001	1.79 (1.4~2.28)	< 0.001	1.85 (1.56~2.2)	< 0.001	1.37 (0.97~1.93)	0.072
**Race**								
White	1	Ref.	1	Ref.	1	Ref.	1	Ref.
Black	1.13 (1.01~1.26)	< 0.001	1.26 (1.00~1.59)	0.049	1.25 (1.04~1.5)	0.019	1.35 (0.97~1.88)	0.076
Others	0.79 (0.71~0.89)	< 0.001	0.89 (0.69~1.14)	0.360	0.78 (0.64~0.95)	0.012	0.71 (0.49~1.03)	0.072
**Marital status**								
Married	1	Ref.	1	Ref.	1	Ref.	1	Ref.
Unmarried	1.14 (1.05~1.24)	0.029	1.05 (0.79~1.40)	0.744	1.06 (0.86~1.32)	0.579	0.89 (0.59~1.34)	0.576
Divorced	1.34 (1.26~1.42)	< 0.001	1.19 (1.00~1.42)	0.048	1.31 (1.16~1.48)	< 0.001	0.95 (0.74~1.22)	0.685
**T stage**								
T1	1	Ref.	1	Ref.	1	Ref.	1	Ref.
T2	1.16 (1.09~1.24)	< 0.001	1.25 (1.08~1.44)	0.003	1.14 (1.02~1.28)	0.021	1.2 (0.97~1.5)	0.094
T3	1.2 (1.060~1.34)	< 0.001	1.58 (1.24~2.01)	< 0.001	1.0 (0.84~1.18)	0.974	1.4 (1.04~1.87)	0.025
T4					1.59 (1.22~2.08)	0.001	3.25 (2.09~5.07)	< 0.001
**PSA**								
< 20	1	Ref.	1	Ref.	1	Ref.	1	Ref.
>20	1.26 (1.16~1.36)	< 0.001	1.75 (1.49~2.05)	< 0.001	1.28 (1.14~1.44)	< 0.001	1.84 (1.5~2.25)	< 0.001
**Gleason score**								
< 8	1	Ref.	1	Ref.	1	Ref.	1	Ref.
8	1.21 (1.10~1.33)	< 0.001	1.82 (1.45~2.28)	< 0.001	1.06 (0.79~1.43)	0.697	0.94 (0.5~1.79)	0.855
9-10	1.52 (1.38~1.68)	< 0.001	3.52 (2.82~4.39)	< 0.001	1.18 (0.93~1.5)	0.167	2.31 (1.44~3.72)	0.001
**Treatment**								
RP	1	Ref.	1	Ref.	1	Ref.	1	Ref.
EBRT	1.62 (1.42~1.86)	< 0.001	1.86 (1.35~2.55)	< 0.001	1.55 (1.29~1.87)	< 0.001	1.58 (1.12~2.21)	0.009

OS: overall survival; PCSS: prostate cancer-specific survival; HR: hazard ratio; Ref: reference; PSA: prostate-specific antigen; RP: radical prostatectomy; EBRT: external beam radiotherapy.
